# Imaging features (CT, MRI, MRS, and PET/CT) of primary central nervous system lymphoma in immunocompetent patients

**DOI:** 10.1007/s10072-018-3669-7

**Published:** 2018-12-22

**Authors:** Gang Cheng, Jianning Zhang

**Affiliations:** Institute of Neurosurgery, Navy General Hospital, Beijing, 100048 China

**Keywords:** Primary central nervous system lymphoma, Non-Hodgkin lymphomas, Imaging

## Abstract

**Background:**

Because of the low incidence of primary central nervous system lymphoma (PCNSL) in non-HIV individuals and because of the lack of specific clinical manifestations and auxiliary examinations, the disease is easily missed or misdiagnosed.

**Objective:**

To analyze the imaging features of PCNSL in non-HIV patients.

**Methods:**

This was a retrospective study of patients with PCNSL treated between January 2001 and December 2011 at the Naval General Hospital (Beijing, China). All included patients were pathologically diagnosed with PCNSL. Specimens were obtained by stereotactic biopsy and diagnosed by pathological examination. Serological panel had to be negative for HIV.

**Results:**

Out of the 118 patients, 73 (61.9%) were male and 45 (38.1%) were female. Median age was 54 (range 11–83) years. All patients had B cell lymphoma. The lesions showed slightly hyperintense shadows on computed tomography (CT) images, and mostly hyperintense T1 and iso- or hyperintense T2 signals on magnetic resonance imaging (MRI). Most lesions showed patchy enhancement after enhanced scanning, and some had the characteristic “butterfly sign” on enhanced MRI. The magnetic resonance spectroscopy of PCNSL manifested as increased Cho peak, moderately decreased NAA peak, and slightly decreased Cr peak. Positron emission computed tomography indicated high metabolism of ^18^F-FDG in PCNSL lesions.

**Conclusion:**

MRI is important in the diagnosis of PCNSL. Understanding the imaging features of PCNSL will help improve its diagnosis in clinics.

## Introduction

Primary central nervous system lymphoma (PCNSL) is a rare form of non-Hodgkin lymphoma (NHL). The disease has an annual incidence of 0.46/100,000 and accounts for 1–2% of all cases of NHL [1] and 1–3% of all central nervous system (CNS) tumors [1, 2]. Compromised immune status is the only known risk factor for PCNSL [3, 4] and the incidence of PCNSL is high in HIV-positive populations [5–7].

The causes of PCNSL in patients with normal immune function are still unknown. Because the incidence of PCNSL is much lower in individuals with normal immune functions compared to the HIV-positive population, there is a lack of large-scale studies. Due to the short course of disease and rapid progression, early diagnosis of PCNSL is very important. Because of the low incidence of PCNSL among non-HIV populations and because of the lack of specific clinical manifestations and auxiliary examinations, the disease is easily missed or misdiagnosed.

A better understanding of the imaging characteristics of PCNSL is critical to improve early diagnosis and treatment, and thus the prognosis of the disease. Therefore, the present study aimed to analyze the imaging features of PCNSL in non-HIV patients.

## Materials and methods

### Study design and patients

This was a retrospective study of patients with PCNSL treated between January 2001 and December 2011 at the Naval General Hospital (Beijing, China). The study was approved by the Ethic Committee of the hospital. The need for individual consent was waived by the committee because of the retrospective nature of the study.

All included patients were pathologically diagnosed with PCNSL. Specimens were obtained by stereotactic biopsy and diagnosed by pathological examination. Serological panel had to be negative for HIV.

### Data collection

Patient data including gender, age, time from first symptoms to diagnosis, clinical symptoms and signs, imaging data of the tumor (including magnetic resonance imaging (MRI), computed tomography (CT), and positron emission computed tomography (PET/CT)), and pathological examinations were collected from the medical charts.

### Imaging

CT was performed using a Philips 256 Slice iCT scanner (Philips, Best, The Netherlands) at 240 mA, 120 kV, 5 mm/slice, scanning speed of 1 slice/s, W80 HU, and C4050 HU. MRI was performed using a Siemens 3.0-T MRI (Siemens, Erlangen, Germany) with a maximum gradient strength of 45 mT/m and a maximum slew rate of 200 mT/m/s. The transmitting and receiving coils were 12-channel standard head-array coils. PET/CT was performed using a GE multifunctional whole-body PET/CT scanner, with PET acquisition parameters of FOV = 256 × 256 mm, array of 128 × 128, slice thickness of 2 mm, and time of 10 min, while CT scanning parameters were set as 120 kV, 150 mA, slice thickness of 3.2 mm, and pitch of 0.938. CT data were used for attenuation correction of PET images, and cross section images were obtained by 3D LOR-RAM LA reconstruction.

### Statistical analysis

Statistical analysis was performed using SPSS 16.0 (IBM, Armonk, NY, USA). Continuous data were presented as mean ± SD. Categorical data were presented as frequencies and analyzed using the Fisher’s exact test. Two-sided *P* values < 0.05 were considered to be statistically significant.

## Results

### Clinical features

All 118 patients had B cell PCNSL. There were 73 males (61.9%) and 45 females (38.1%) (ratio of 1.62:1). Age at diagnosis ranged from 11 to 83 years (median, 54 years). Among the males, four patients were 11–20 years of age (5.5%), four were 21–30 (5.5%), nine were 31–40 (12.3%), 11 were 41–50 (15.1%), 17 were 51–60 (23.3%), 18 were 61–70 (24.7%), and 10 were ≥ 71 (13.7%). Among the females, three patients were 21–30 years of age (6.7%), seven were 31–40 (15.6%), six were 41–50 (13.3%), 15 were 51–60 (33.3%), 10 were 61–70 (22.2%), and four were ≥ 71 (8.9%). There was no significant difference in age between male and female patients (*P* = 0.616).

The time from the first symptoms to diagnosis ranged from 7 days to 3 years (median, 28 days) (Table 1). The initial symptoms and signs included headache, dizziness and nausea (51.1%), limb dysfunction (47.7%), memory loss (13.9%), visual impairment (12.8%), barylalia (12.8%), somnolence (8.1%), fatigue (5.8%), slow response (4.6%), cerebellar symptoms (4.6%), and psychiatric symptoms (2.3%) (Table 1).

**Table 1:** Characteristics of the patients

	Values
*N*	118
Males	73, 61.9%
Females	45, 38.1%
Age (years)	Median 54, range 11–83
11–50	44, 37.3%
> 50–70	60, 50.8%
> 70	14, 11.9%
Time from first symptoms to diagnosis	Median 28 days, range 7 days to 3 years
< 3 months	78.8%
< 1 month	57.5%
Initial symptoms and positive signs	
Headache, dizziness, and nausea	51.1%
Limb dysfunction	47.7%
Memory loss	13.9%
Visual impairment	12.8%
Barylalia	12.8%
Somnolence	8.1%
Fatigue	5.8%
Slow response	4.6%
Cerebellar symptom	4.6%
Psychiatric symptoms	2.3%

Lesion distribution (multiple lesions were counted into their corresponding anatomical sites, and the total number of lesions is then > 118) included 21 lesions in the left frontal lobe, 24 in the right frontal lobe, 10 in the left parietal lobe, 15 in the right parietal lobe, 8 in the left temporal lobe, 9 in the right temporal lobe, 5 in the left occipital lobe, 2 in the right occipital lobe, 34 in the basal ganglia, 26 in the thalamus, 19 in the corpus callosum (mostly in the splenium of corpus callosum), 13 in the lateral ventricle, 6 in the cerebellum, and 19 in the brain stem. Among the 118 patients, 63 (53.4%) had a single lesion, including 58 supratentorial lesions (14 in the frontal lobe, 10 in the parietal lobe, 3 in the temporal lobe, 2 in the occipital lobe, 13 in the basal ganglia, 7 in the thalamus, 5 in the corpus callosum, and 4 in the lateral ventricle) and 5 subtentorial lesions (3 in the cerebellum and 2 in the brain stem). The remaining 55 patients had multiple lesions (46.6%) (Table 2).

**Table 2:** Locations of the lesions

Locations	Number	Percent
Cerebral hemispheres	94	79.6%
Basal ganglia region	34	28.8%
Thalamus	26	22.0%
Corpus callosum^a^	19	16.1%
Lateral ventricle	13	11.0%
Cerebellum	6	5.1%
Brainstem	19	16.1%
Multiple lesions	55	46.6%
Single lesions	63	53.4%

Patients could have multiple lesions and each lesion was counted

^a^Most lesions were distributed in the splenium of the corpus callosum

### CT features

**Fig. 1:**
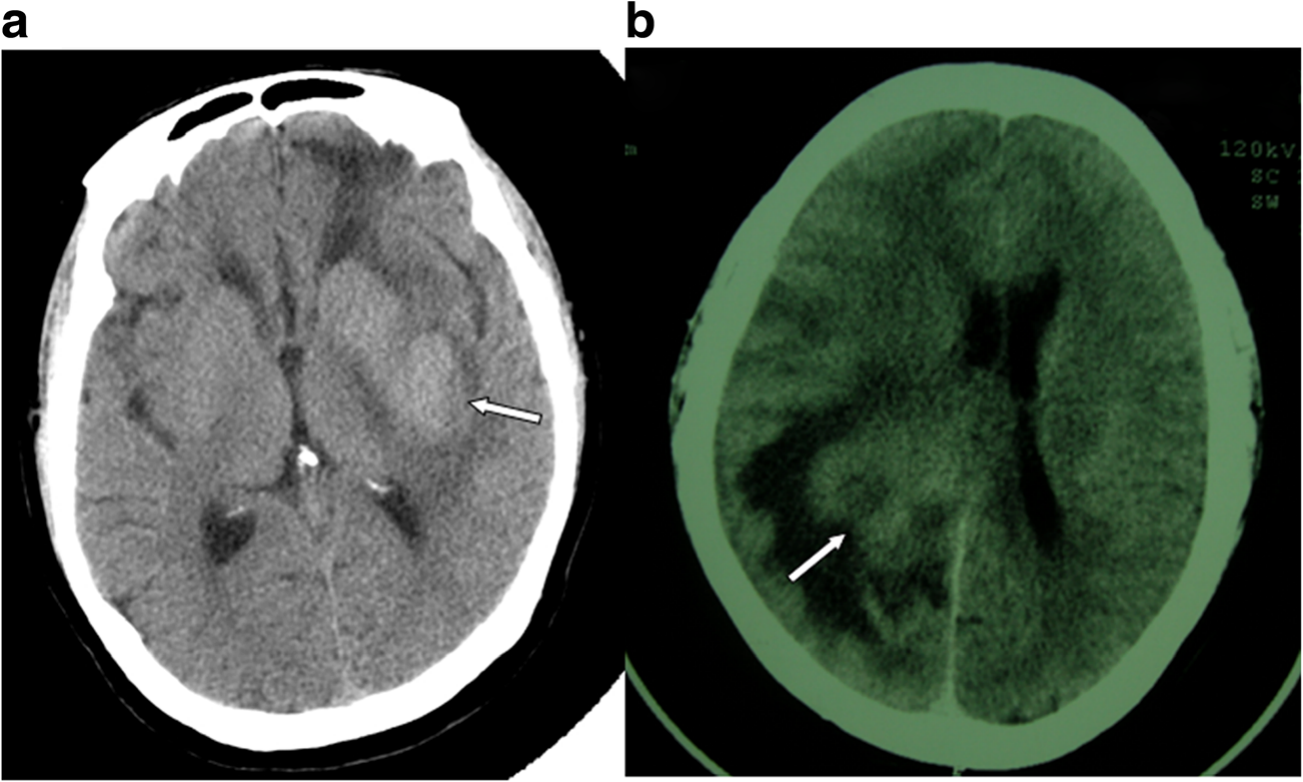
**a** CT features of primary central nervous system lymphoma (PCNSL) (arrow). **b** Obvious peritumoral edema (arrow)

CT examination showed that most lesions were space-occupying lesions with slightly higher intensity. Irregular patchy enhancement was shown after enhanced scanning. Since PCNSL has a very high nuclear/cytoplasmic ratio, most lesions showed high density shadows on CT scanning, but shadows of low, equal, slightly higher, or even mixed density could also be seen, and would most likely develop irregular patchy enhancement. On CT images, mild edema could be seen in peritumoral areas (Fig. 1), but some lesions might show significant peritumoral edema.

### MRI features

**Fig. 2:**
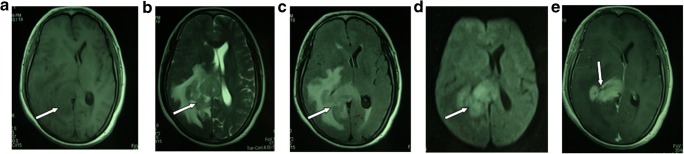
Male, 47 years of age, 1-month history of mental abnormalities. The arrow points to the typical magnetic resonance imaging (MRI) features of primary central nervous system lymphoma (PCNSL). **a** T1-weighted imaging. **b** T2-weighted. **c** Fluid attenuation inversion recovery (FLAIR). **d** Diffusion-weighted imaging (DWI). **e** Significant enhancement of the lesion on Gd-DTPA MRI with a typical “belly button” sign

**Fig. 3:**
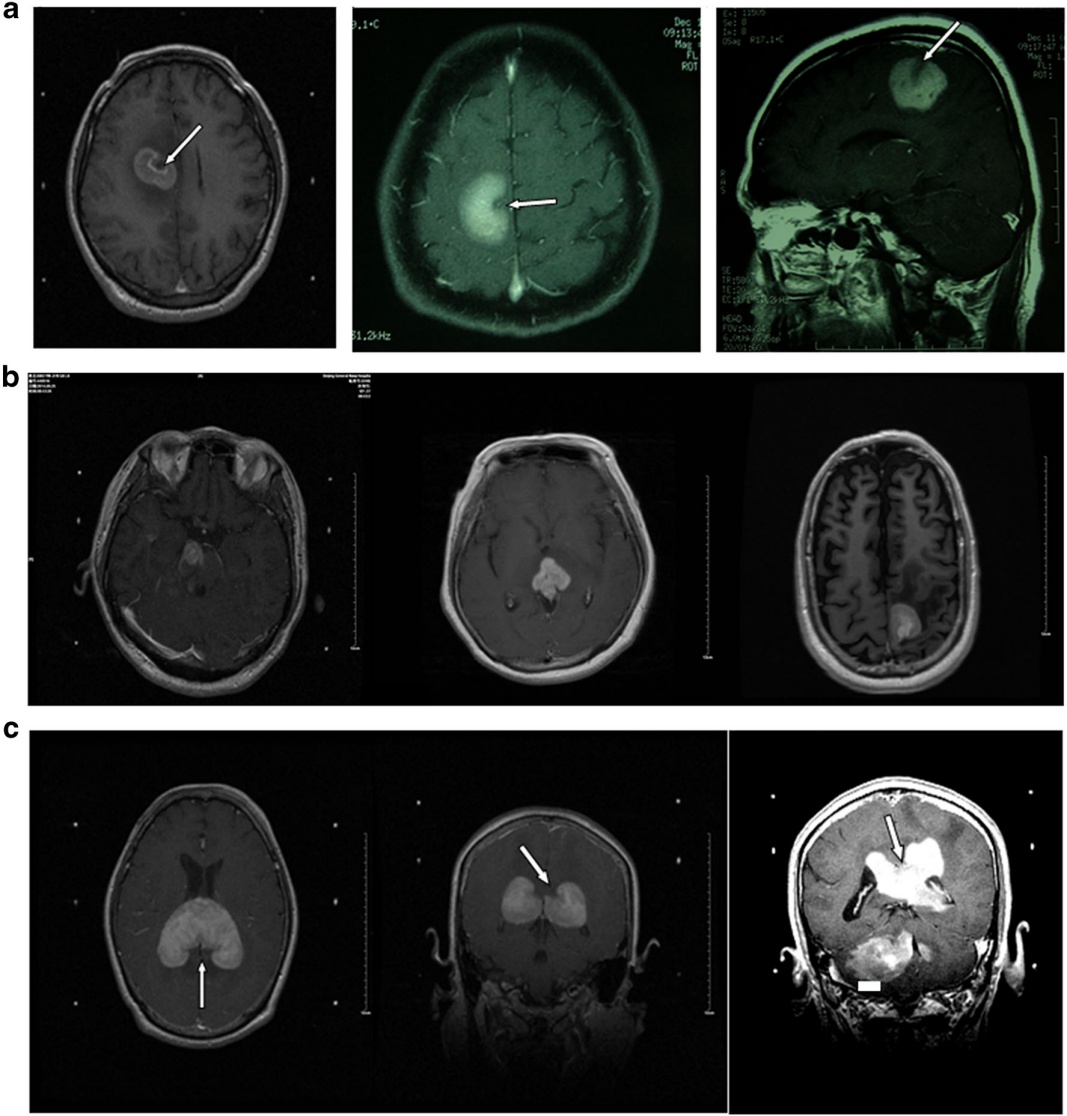
**a** The typical “belly button” sign of primary central nervous system lymphoma (PCNSL) on magnetic resonance imaging (MRI) (arrow). **b** The “snow ball” sign of primary central nervous system lymphoma (PCNSL) on MRI. **c** The “butterfly sign” of primary central nervous system lymphoma (PCNSL) on MRI

**Fig. 4:**
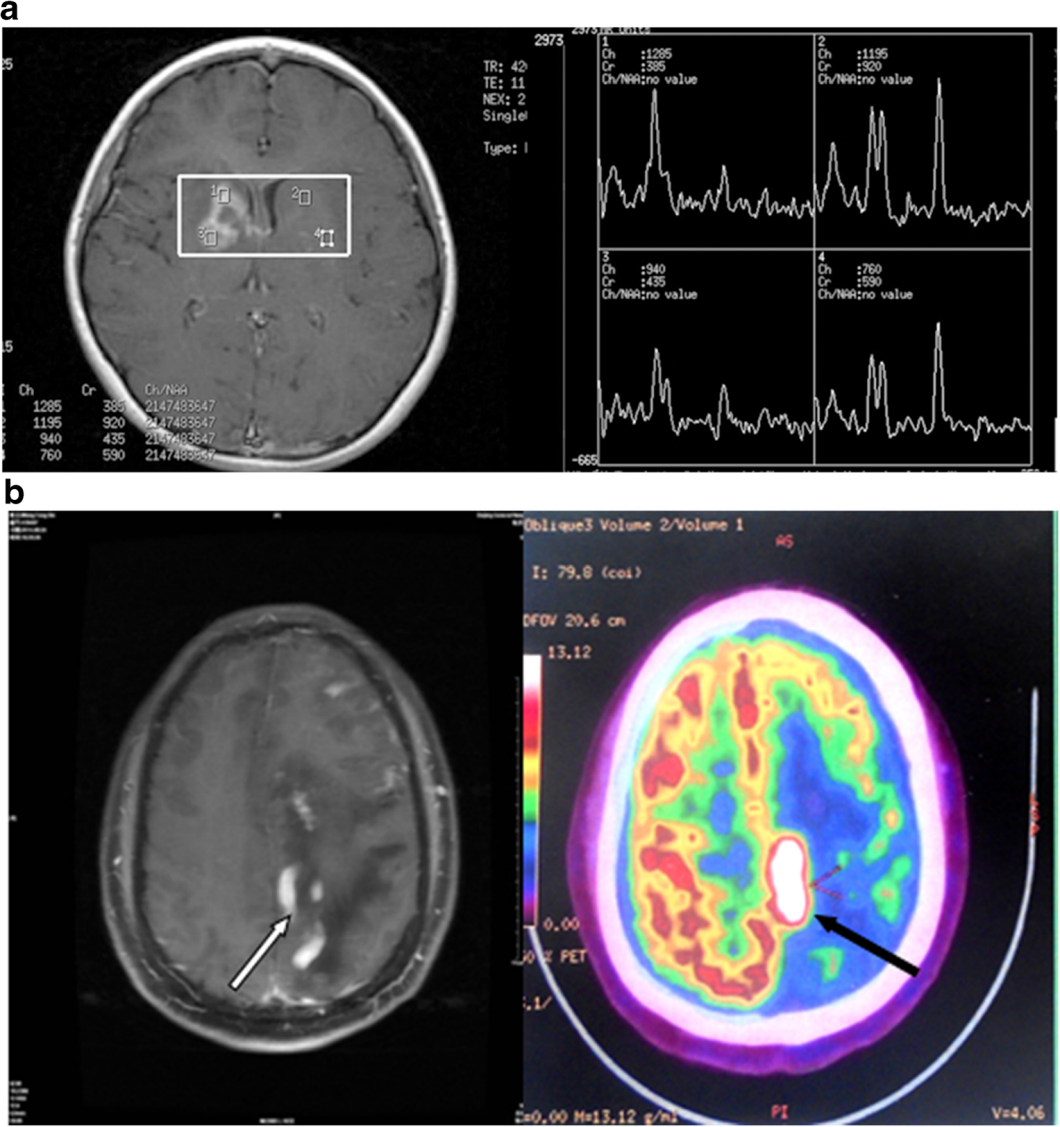
**a** Female, 35 years of age, 2-month history of headache and aggravation for 1 week. Magnetic resonance spectroscopy (MRS) of primary central nervous system lymphoma (PCNSL). **b** Male, 41 years of age, history of memory loss for 1 month. FDG-PET imaging of PCNSL. (Left) Lesions on Gd-DTPA magnetic resonance imaging (MRI) (white arrow). (Right) Significantly high metabolism of the lesions on FDG-PET (black arrow)

MRI examination was mainly based on hyperintense T1-weighted (T1WI), iso- or hyperintense T2-weighted (T2WI), and high fluid attenuation inversion recovery (FLAIR) images for PCNSL lesions. Enhanced scanning showed significant patchy enhancement; some cases had “incision,” “angular,” “fist,” or “hard ring” signs. MRI of PCNSL would show iso- or slight hypointensity on T1WI and iso- or slight hyperintensity on T2WI, whereas most tumors would show hypo-intensity on T2WI. Almost all patients had uniform or nodular enhancement after enhanced scanning (Fig. 2). Since lymphomas are less likely to have hemorrhage or necrosis, ring-enhancement was rarely seen. The lesions had irregular morphologies, with either obvious or unobvious edema at the periphery, few of which causing midline displacement or intraventricular compression. The image had high FLAIR signal and enhanced scanning showed significant patchy enhancement. Some cases had the “belly button,” “angular,” “snow ball,” or “hard ring” signs (Fig. 3a, b) all of which would be helpful to diagnose PCNSL [8–10]. Apart from these, if the lesion mainly invaded the midline, starting from one side and across the corpus callosum to the contralateral side, with simultaneous involvement of both corpus callosum and frontal lobe, then there would be a typical “butterfly sign” (Fig. 3c). The lesions were also accompanied by peritumoral edema, either patch-like, flame-like, or finger-like, but the size of the tumor was not related to the level of edema. Magnetic resonance spectroscopy (MRS) of PCNSL showed increased Cho peak, moderately decreased NAA peak, slightly decreased Cr peak, and significantly high Lip peak (Fig. 4a).

### PET/CT

PDG-PET examination of PCNSL indicated significantly high metabolism of ^18^F-2-fluro-D-deoxy-glucose (^18^F-FDG) in PCNSL lesions (Fig. 4b).

## Discussion

PCNSL is less common among people with normal immune function than patients with a compromised immune status [3, 4], and few large-scale studies are available, especially in China. Because of the low incidence of PCNSL among non-HIV populations and because of the lack of specific clinical manifestations and auxiliary examinations, the disease is easily missed or misdiagnosed. Therefore, the present study aimed to analyze the imaging features of PCNSL in non-HIV patients. The results showed that MRI is of important significance in the diagnosis of PCNSL. Understanding the imaging features of PCNSL would help its diagnosis in clinics.

In the present study, the mean age of onset was 53.4 ± 15.9 years, and more than half of the patients were between 51 and 70 years of age. This is supported by some Chinese studies [11, 12], and a European study showed a mean age at diagnosis younger by 10 years compared with the present series [13]. Whether the difference is associated with ethnicity needs to be supported by more evidence. Besides, in the present study, about 30% of the patients were 31–50 years of age, underlining that the onset age of PCNSL has a wide range, which was nonetheless similar between men and women.

The reasons for the development of PCNSL in people with normal immune function are currently unknown. PCNSL might be derived from systemic malignant diseases that have been eliminated by the immune system, but neoplastic B cells can escape this elimination due to the protection of CNS, which could explain why most PCNSL are B cell lymphoma. Indeed, 90% of PCNSL patients with normal immune function have diffuse large B cell lymphoma and most cells are from the germinal center [11]. Other common pathological types include immunoblastic lymphoma and lymphoblastic lymphoma; less than 4% of PCNSL are T cell lymphoma [1, 2, 5, 12]. In this study, all patients had B cell lymphoma.

The prognosis of PCNSL is poorer than that of systemic lymphoma of the same pathological type [1, 2]. In the present series, the average time from the first symptoms to diagnosis was 3.5 ± 6.7 months, and 57.5% of patients had a disease course of < 1 month. Clinical symptoms of PCNSL are associated with the mass effect of the tumor as well as lesion location. Common sites include the cerebral hemisphere, basal ganglia, and corpus callosum [9], and most patients have lesions in the central hemisphere or periventricular white matter [13]. Based on a previous study, 20–43% of PCNSL lesions are in the frontal lobe, 13–20% in the basal ganglia, 9–13% in the brain stem and cerebellum, and only 1–2% in the spinal cord [2]. Another study suggested that most patients (60–80%) with normal immune function have a single PCNSL lesion [14]. As reported by Kuker et al. [9], 65% of the patients had a single lesion that was mainly distributed in the cerebral hemisphere (36.4%) and corpus callosum (27.7%). Haldorsen reviewed 75 patients with PCNSL and normal immune function [2], and their data showed that 45% were simple lesions; 43% of the lesions were in the frontal lobe, 56% were around the ventricles, and 13% could not be clearly identified. In the present study, the lesions were mainly distributed in the deep and periventricular structures of the brain. But comparing to the above studies, our series shows a higher incidence of multiple lesions (47%). Because the Navy General Hospital has the leading position of stereotactic biopsy in China, many suspected cases of PCNSL are referred for definitive diagnosis, probably increasing the frequency of multiple lesions. This might be the reason for the high frequency of multiple lesions in this study, and it also indicates that multiple lesions are not rare among patients with PCNSL with normal immune function. Another difference was the lesion distribution. Many single lesions were distributed in the basal ganglia and thalamus (31.7%) and only 22.2% were distributed in the frontal lobe, but we are unsure about whether such difference is of clinical significance. Different distribution of lesions could also affect the patients’ clinical symptoms. Intracranial hypertension was the most common symptom in our study, and the incidence of limb dysfunction was also high (47.7%). This might be associated with the lesions being distributed in the basal ganglia. Since the percentage of lesions located in the corpus callosum and frontal lobe was lower than that reported by previous studies, the incidence of change of cognition, behavior, and personality was also low compared to previous studies [3, 15, 16].

In the present study, CT scanning of PCNSL showed lesions with hyper- or iso-intensity, and enhanced scanning displayed significant enhancement. Nevertheless, compared with other intracranial space-occupying diseases, CT examination of PCNSL suggested no differences, as suggested by previous studies [13, 17–19]. MRI is the main imaging methods for PCNSL diagnosis and the imaging features of PCNSLs are closely related to their histopathology. Indeed, PCNSL tissues are with high cell density, high nuclear/cytoplasmic volume ratio, enriched in reticular fibers, and small stromal components. Therefore, they will show iso- or hypointensity on T1WI, and iso- or relative hyperintensity, or even hypointensity on T2WI. PCNSL would also develop characteristic “incision,” “angular,” or “fist” signs on enhanced MRI [13, 17–20], which would be helpful for the diagnosis of PCNSL. Apart from these, linear enhancement along the periventricular area strongly suggests PCNSL [21]. These imaging features could be attributed to that during the “angiotropic” invasive growth of tumor, tumor cells arrange themselves centripetally around the Virchow–Robin space, and show a sleeve-like infiltration pattern. The heterogeneity of peripheral tissues and vascular occlusion would also cause heterogeneous growth of tumor, and this might possibly explain why cystic degeneration and necrosis is less common in PCNSL, and ring-enhancement is also rarely seen [13, 17–20]. Since the white matter of the brain has a loose structure, PCNSL could infiltrate via the Virchow–Robin space of perforating arteries into the midline region, including deep white matter and periventricular areas, and it could also progress across the lobes into the contralateral white matter via the corpus callosum, thereby showing the typical “butterfly sign”.

Since routine contrast enhanced MRI only reflects the damage to the blood brain barrier and not the degree of tumor angiogenesis, the use of DWI would be useful, as previous studies reported that DWI could accurately reflect the level of tumor angiogenesis [22]. A previous study observed an inverse correlation among cellular density, apparent diffusion coefficient (ADC) values, and survival [23]. The ADC is used to describe the diffusion of water molecules [24]. Microscopic observation of PCNSL suggested densely distributed tumor cells, narrowed extracellular spaces, and high nuclear/cytoplasmic volume ratio, all of which would limit the diffusion of water molecules in the tumor, resulting in low ADC value and high DWI signal. The ACD value of PCNSL is normally within 0.7–0.9 × 10^−3^ mm^2^/s, which is lower than that of astrocytoma, glioblastoma multiforme (GBM), and cerebral gliomatosis [25]. About 90% of PCNSL patients have restricted water diffusion in the lesions before treatment, but there are still some lesions with unrestricted water diffusion [12].

N-acetyl-L-aspartic acid (NAA), choline (Cho), creatine (Cr), lactate (Lac), and lipids (Lip) are the major metabolic substances in the brain. NAA is mainly present in mature neurons, and its content reflects the functional status of the neurons, so that in normal brain, the NAA peak would be the highest on the ^1^H-MRS images [26, 27]. For craniocerebral diseases with neuron damage, the NAA peak will reduce and even disappear in brain tumors without neurons such as meningioma and metastatic tumor. Cho is the marker of cell membrane transition, which reflects the proliferation of cells [26, 27]. The Cho peak would be increased in almost all primary and secondary brain tumors [27]. Cr is the most stable metabolite in the brain and is negatively associated with energy metabolism [26]. The occurrence of the Lip peak indicates tumor necrosis [27]. The manifestations of PCNSL include increased Cho peak, moderately decreased NAA peak, and slightly decreased Cr peak, which indicate neuron damage and increased tumor cell proliferation [8, 28, 29]. The significantly high Lip peak differentiates lymphoma from other solid tumors, and some scholars believe that it is caused by massive phagocytosis of free fatty acids by macrophages [30]. The huge Lip peak could be considered as a characteristic sign of lymphoma [29].

Single photon emission computed tomography (SPECT) and PET can distinguish PCNSL from other common lesions in AIDS patients [31]. Zou et al. [32] showed that PET-CT was sensitive for PCNSL. In addition, PET-CT could determine the sensitivity of PCNSL to chemotherapy faster than MRI [33]. Nevertheless, the uptake rate of FDG is relatively high in the basal ganglia, thalamus, and gray matter [34]; thus, the location of PCNSL might be incorrect. For uncharacteristic PCNSL lesions, if MRI suggests unobvious or diffuse lesions, FDG-PET may not improve lesion localization nor increase positive diagnosis [35]. On the other hand, the brain tissue has a very low uptake rate of ^11^C-methionine; thus, the development of ^11^C-methionine-PET could be more significant than FDG-PET for PCNSL. PET could help identify lesions that CT or MRI failed to detect and could also be used in assessing treatment efficacy.

Of course, the present study is not without limitations. The sample size was small and from a single institution. In addition, the study period spanned a long time the technology and experience of radiologists changed during this period. Finally, the study was retrospective, limiting the data to that available in the medical charts.

## Conclusions

In conclusion, PCNSL is a rare malignant tumor with diverse clinical symptoms, and it is easily missed or misdiagnosed. Understanding the imaging features of PCNSL would help to early diagnose and screen for PCNSL, thereby improving the rate of PCNSL diagnosis and facilitating early treatment of this disease. CT and conventional MRI showed no features characteristic to PCNSL, while DWI, MRS, and PET/CT are promising.
